# Considerations and prospects on the role of antecubital approach in adrenal venous sampling for primary aldosteronism: a correspondence

**DOI:** 10.1097/JS9.0000000000003330

**Published:** 2025-09-05

**Authors:** Yunyi Yang, Zheng Yao, Hongjie Yang

**Affiliations:** Yueyang Hospital of Integrated Traditional Chinese and Western Medicine, Shanghai University of Traditional Chinese Medicine, Shanghai, China

**HIGHLIGHTS**

1. **Antecubital Approach Reduces Adrenal Vein Rupture Risk**:

The antecubital approach significantly lowers the risk of adrenal vein rupture during adrenal venous sampling (AVS), a rare but serious complication. Further studies are needed to explore the pathophysiological mechanisms, including the impact of anatomical variations and catheter design.

**Need for Comprehensive Patient Subgroup Analysis**:

While the antecubital approach shows benefits for obese patients, the study overlooks other factors, such as age, sex, BMI, and comorbidities like diabetes, which may influence AVS outcomes. Detailed subgroup analyses and advanced imaging techniques are necessary to optimize patient-specific treatment strategies.

**Consideration of Cost-Effectiveness and Radiation Exposure**:

The time-saving benefits of the antecubital approach are promising, but its cost-effectiveness and radiation exposure remain underexplored. Future studies should assess these factors to ensure that the benefits outweigh potential risks and justify broader adoption, especially in resource-limited settings.

*Dear Editor,*

We read with great interest the article entitled *“Antecubital versus femoral approach for adrenal venous sampling in the patients with primary aldosteronism: a randomized controlled trial”*^[1]^, published in the *International Journal of Surgery*. The authors’ evaluation of the antecubital approach for adrenal venous sampling (AVS) in primary aldosteronism (PA) demonstrates promising results in terms of success rates, safety, and procedure duration. However, we believe several aspects warrant further investigation to enhance understanding and guide future research. This study is compliant with the TITAN Guidelines 2025^[2]^.

First, the study reported that the antecubital approach significantly reduced the incidence of adrenal vein rupture, a rare but serious complication during AVS. This may be due to avoiding excessive catheter insertion, a common issue with the femoral approach. However, the pathophysiological mechanisms behind this reduction remain insufficiently explored. It is unclear whether the reduced rupture rate is due to technical differences or if anatomical variability and catheter selection play a role^[3]^. Previous research suggests that excessive catheter insertion, especially with reverse-curve catheters (e.g., Simmons type 2), may increase rupture risk^[4]^. The lower risk with the antecubital approach could be related to minimized insertion depth or anatomical factors, such as the alignment of the right adrenal vein and catheter shape^[5]^. While the MPA1 catheter is suitable for most antecubital cases, its effectiveness in patients with anatomical variations, such as vascular anomalies, remains uncertain^[6]^. Anatomical anomalies challenge AVS and may affect success rates for both femoral and antecubital access. Future research should focus on alternative catheter designs for patients with complex anatomy, such as microcatheters for more precise sampling from the left adrenal vein^[7]^. Tailoring catheter selection and techniques to individual anatomy will optimize procedures and reduce complications.

Second, while the study highlighted the antecubital approach’s advantage in reducing complications among obese patients, it did not fully consider other factors – such as sex, age, BMI, hypertension, and renal insufficiency – that could influence outcomes^[8,9]^. Relying solely on BMI may overlook other contributors, especially in high-risk groups like those with hypertension or hypokalemia^[10]^. The impact of comorbidities, such as diabetes, should also be considered. Diabetes can promote intimal hyperplasia, reduce vascular elasticity, and worsen microcirculatory issues, increasing the risk of AVS failure and complications like thrombosis or infection. Metabolic abnormalities in diabetic patients may complicate the procedure, increasing venous cannulation difficulty and the risk of complications. Future studies should include subgroup analyses of sex, age, BMI, and comorbidities like diabetes to evaluate their impact on AVS success and complications. Additionally, incorporating advanced imaging techniques, such as preoperative ultrasound, could help identify the optimal access route for each patient. Perioperative management strategies (including antiplatelet therapy, fluid supplementation, or vasoactive agents) aimed at reducing vascular complication risk should also be prioritized, particularly in patients with underlying disease. A thorough exploration of these variables will enable the development of more individualized treatment strategies, thereby optimizing AVS outcomes and ensuring procedural safety and efficacy.

Third, the authors discussed the time-saving advantage of the antecubital approach, particularly in terms of procedural and hospitalization duration, which facilitates more efficient resource utilization – especially in resource-limited settings. However, the study did not adequately address cost-effectiveness or radiation exposure. While shorter procedure times are beneficial, whether this translates into substantial cost savings warrants evaluation. Although the antecubital approach may reduce arterial puncture and hematoma risk, it may also introduce other complications, such as catheter-related infections or complex catheter manipulations, which could offset the time savings. Furthermore, radiation exposure remains a concern, particularly when imaging guidance is required, which may result in additional exposure. Future studies should evaluate radiation exposure to ensure no harm to patients or operators. If the antecubital approach requires extra training or equipment, these factors should also be incorporated into cost-effectiveness assessments. As the authors noted, the cost-effectiveness of AVS remains a matter of debate. Comprehensive economic analyses are needed to determine whether the reductions in procedure and hospitalization time are sufficient to offset other costs, thereby providing a basis for the broader adoption of the antecubital approach.

In conclusion, this study provides valuable insights into the potential benefits of the antecubital approach for AVS in PA patients. However, further research is required to investigate the long-term impacts of catheter selection, anatomical variation, and the cost-effectiveness and radiation concerns of the technique. Larger multicenter studies with detailed subgroup analyses are essential to validate these findings and guide clinical practice. Such efforts will help establish the antecubital approach as a viable alternative to femoral access in AVS.

## Data Availability

Not applicable.
